# Anaesthesia Considerations in a Case of Alkaptonuria Posted for Total Knee Arthroplasty

**DOI:** 10.7759/cureus.61882

**Published:** 2024-06-07

**Authors:** Saurabh M Barde, Pratibha U Deshmukh, Mukesh Laddha, Dilip Rathi

**Affiliations:** 1 Anaesthesia, Datta Meghe Medical College, Datta Meghe Institute of Higher Education and Research, Nagpur, IND; 2 Orthopaedics, RNH Hospital Pvt. Ltd., Nagpur, IND

**Keywords:** ultrasound guidance, adductor canal block, videolaryngoscopy, homogentisic acid, ochronosis, alkaptonuria

## Abstract

Alkaptonuria is a rare hereditary condition in which homogentisic acid is deposited in collagenous tissues, leading to blackish discoloration, degenerative changes, restricted mobility, and pain in the affected part. The skeletal system is commonly affected, resulting in the stiffening of the vertebral spine, shoulders, knees, hip joints, and thoracic cage. Additionally, the degenerative process involves heart valves, endocardium, and kidneys, with associated pathophysiological changes. These patients present significant challenges in neuraxial anesthesia, airway management, and postoperative pain relief. In this report, we present the anesthetic management of a case of alkaptonuria undergoing total knee arthroplasty and discuss the encountered difficulties. We conclude that the perioperative anesthesia management of alkaptonuria patients requires thorough planning to effectively address the various challenges associated with the administration of anesthesia.

## Introduction

Alkaptonuria, a hereditary condition characterized by a deficiency of homogentisate 1,2 dioxygenase (HGD), results in the deposition of homogentisic acid (HGA) in various collagenous tissues throughout the body [1]. This rare autosomal recessive disorder affects the metabolism of tyrosine, with HGD playing a crucial role in converting HGA into maleylacetoacetic acid. The global incidence of alkaptonuria ranges from 1:250,000 to 1:1,000,000 in live births, though specific incidence data for the Indian population are currently unavailable [2]. Excess HGA is initially excreted by the kidneys, but over time, it gradually accumulates in collagenous tissues.

Alkaptonuria is characterized by distinctive clinical features, including the dark discoloration of urine upon exposure to sunlight, ochronosis (a bluish-black discoloration of tissues), and arthritis [3]. Degenerative changes manifest throughout the entire spine, with the lumbar region being the most commonly affected. These changes include intervertebral disc calcifications, narrowed disc spaces, ligament calcification, and the formation of osteophytes, resulting in significant lumbar spine stiffening akin to ankylosing spondylitis [4]. Involvement of the cervical spine restricts neck extension. These structural alterations pose challenges in neuraxial (spinal, epidural) anesthesia and airway management. Additionally, individuals with alkaptonuria may experience restrictive lung disease, valvular heart involvement, kidney stones, scleral discoloration, ear thickening, and arthritis affecting one or more major joints. Thorough preoperative evaluation and meticulous planning are crucial for the successful perioperative anesthetic management of individuals with alkaptonuria.

## Case presentation

A 62-year-old male patient (American Society of Anesthesiologists physical status - 2) presented with a history of severe pain in both knee joints, accompanied by restricted movements and difficulty in standing, walking, and using western toilets. The symptoms had progressed, and their severity had increased over the last two months. The patient also reported an inability to bend forward and experienced very restricted, painful side movements of both upper limbs at the shoulder joint. The patient had a confirmed diagnosis of alkaptonuria, with a history of dark urine coloration over time. The patient's mother had a similar diagnosis with milder symptoms and had undergone hip arthroplasty under spinal anesthesia at the age of 82, four years before her demise. The patient occasionally used tablet (tab) ibuprofen 400 mg stat when suffering from severe pain and regularly took Tab paracetamol 650 mg as and when required for pain relief. Strong painkillers were avoided due to acid peptic issues. The patient was scheduled for left-sided total knee arthroplasty as it was the more painful joint.

**Figure 1:**
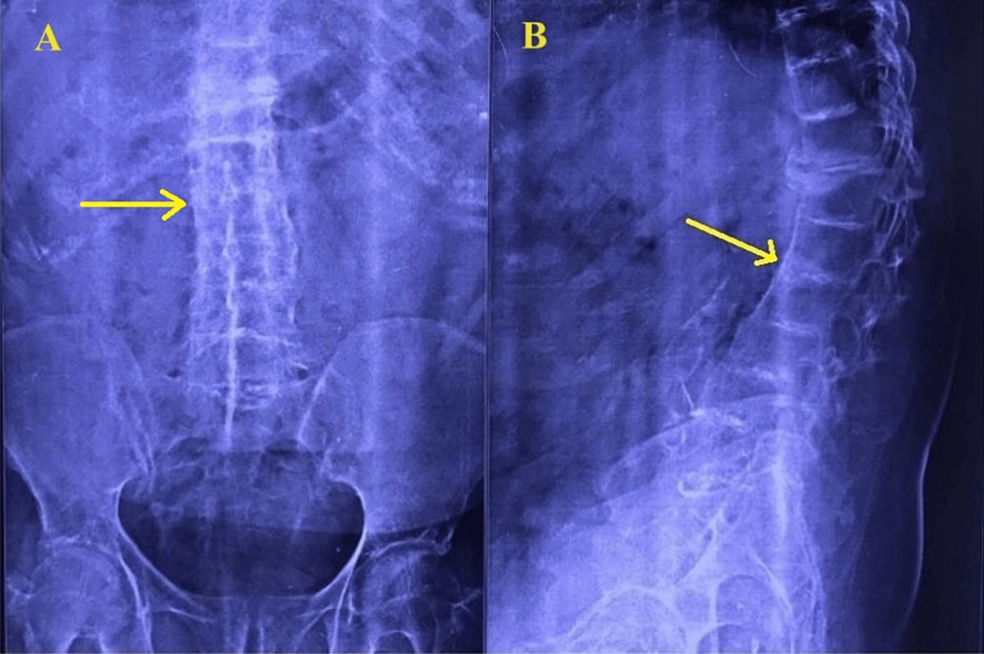
X-ray spine A: Antero-posterior view of the lumber spine; B: Lateral view of the lumber spine. Yellow arrows showing fused vertebrae.

**Figure 2:**
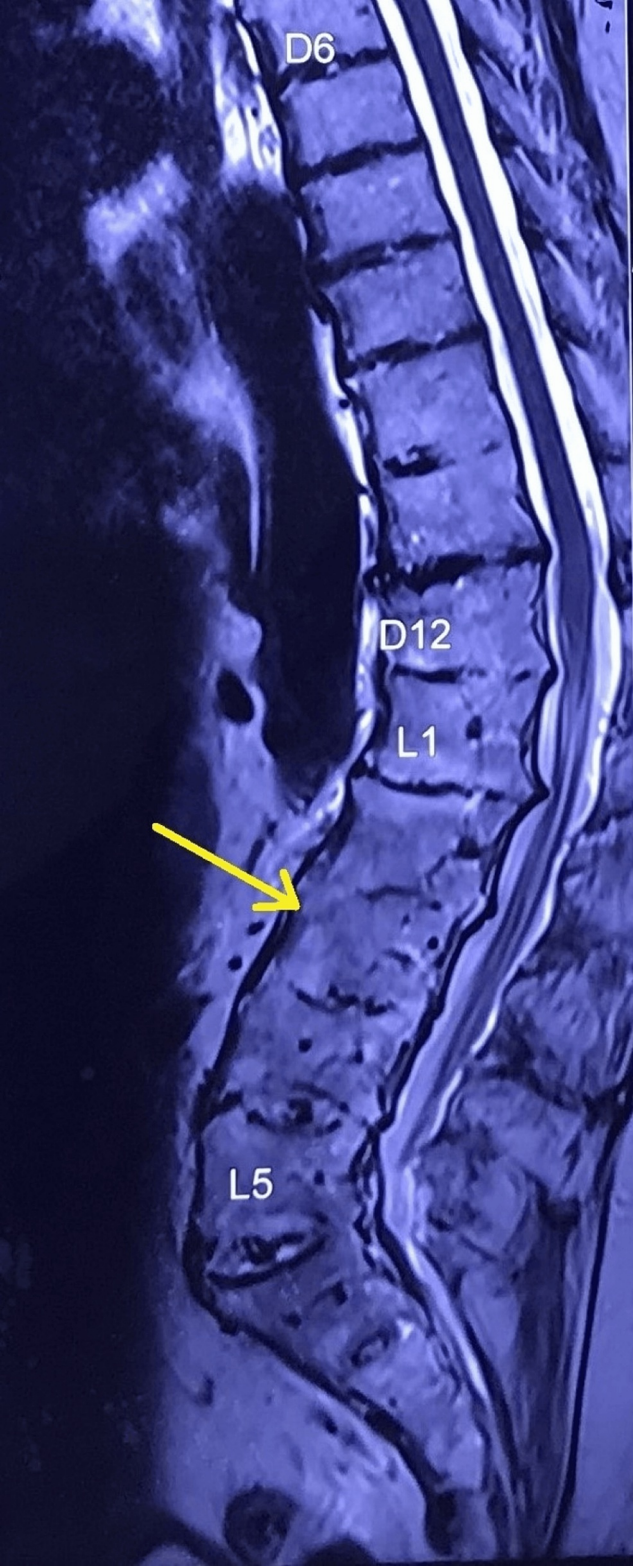
Magnetic resonance imaging (MRI) of the spine D6: Thoracic spine 6th vertebra; D12: Thoracic spine 12th vertebra; L1: Lumbar spine 1st vertebra; L5: Lumbar spine 5th vertebra. Yellow arrows showing fused lumbar vertebrae.

The patient with a body mass index of 30.45 kg/m^2^. Routine home activities were challenging due to pain. On examination, the patient was conscious and alert, with a regular heart rate of 90 beats/minute and blood pressure of 120/70 mm Hg. A cardiovascular examination revealed a murmur. Two-dimensional echocardiography showed degenerative aortic and mitral valve diseases with mild regurgitation at both valves. Left ventricular systolic function was normal with an ejection fraction of 60%, and there was grade one left ventricular diastolic dysfunction. Respiratory system examination and pulmonary function tests were normal. Airway examination revealed a modified Mallampatti grade 3 classification with restricted neck extension. The lumbar spine exhibited increased lordosis and scoliosis with concavity towards the right side. A lumbar spine radiograph confirmed these clinical findings and revealed very narrow interspinous spaces. Spine radiograph and magnetic resonance imaging (MRI) of the spine indicated ossification of D9-10 to L5-S1 intervertebral discs (Figures 1-2), ossification of the anterior longitudinal ligament, and facetal joint ankyloses in the lumbar area. Cervical spine screening showed posterior disc bulges - osteophyte complex from C3 to C7, along with ligamentum flavum hypertrophy and impingement on exiting nerve roots bilaterally. Abdominal ultrasonography revealed non-obstructing calculi in both kidneys with normal blood urea and serum creatinine. Other relevant hematological investigations were normal.

The patient was informed about the anesthesia plan, and necessary informed consent was obtained. Intravenous access was challenging due to difficulty visualizing veins and bilateral pain on shoulder abduction, external rotation, and forearm supination. A 22-gauge cannula was secured on the dorsum of the right hand. In the operating room, monitoring included a five-lead electrocardiogram (ECG), non-invasive blood pressure (NIBP), and pulse oximetry. The lumbar spine was scanned using a low-frequency curved ultrasound probe (BPL® E-Cube 8 LE ultrasound machine; BPL Medical Technologies, Bangalore, India). Due to poor sonography visibility caused by hyperacoustic shadows, a decision for general anesthesia was made.

After adequate pre-oxygenation, general anesthesia was induced using intravenous injection of midazolam 1 mg, fentanyl 100 mcg, and propofol 140 mg titrated as per requirement. Mask ventilation was confirmed, and intravenous cis-atracurium 8 mg was used for endotracheal intubation. Video laryngoscopy (TUORen® Videolaryngoscope VLHM5A; Tuoren Medical Device India Pvt. Ltd., Haryana, India) guided Portex cuffed endotracheal tube number 8.5 used for tracheal intubation. Anesthesia was maintained with an air-oxygen mixture and isoflurane, with controlled ventilation and end-tidal carbon dioxide monitoring.

**Figure 3:**
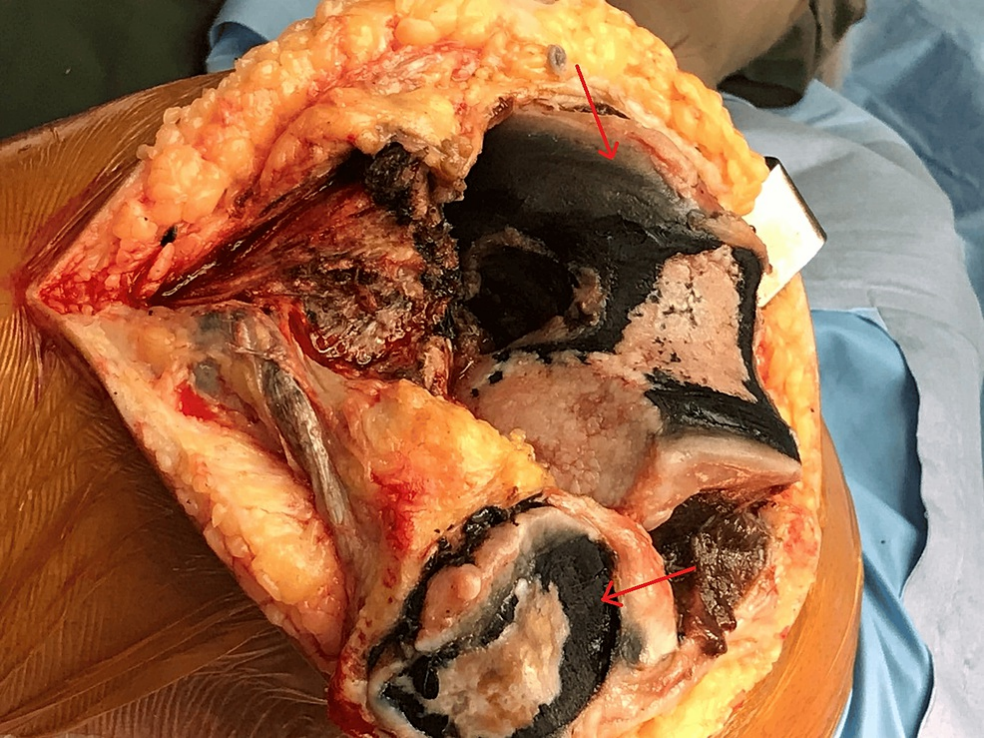
Blackish discolouration of the knee joint Red arrows showing black pigment on bone surface.

A left-sided femoral nerve block was performed under ultrasound guidance using 10 mL of 0.5% injection (Inj) levobupivacaine. After exposing the knee joint, pigmented surfaces were noticed (Figure 3). Robotic-assisted (CORI - Smith & Nephew®) left knee arthroplasty was conducted. The surgeon employed an analgesic cocktail infiltration around the posterior capsule of the knee using 30 mL Inj ropivacaine 0.2%, Inj ketorolac 30 mg, and Inj methylprednisolone 80 mg depot preparation. The surgery lasted for 100 minutes. After completion, an ultrasound-guided adductor canal block was administered with 10 mL 0.2% Inj ropivacaine using a high-frequency linear probe (5-12 MHz; BPL® E-Cube 8 LE ultrasound machine), and a 16-gauge catheter (Portex® epidural) was inserted for post-operative pain management. The trachea was extubated after the complete reversal of the muscle relaxant effect by Inj neostigmine 2.5 mg and Inj glycopyrrolate 0.4 mg. Post-operative analgesia was provided with a continuous adductor canal infusion of 0.2% Inj ropivacaine (3-5 mL/hour) and Inj paracetamol 1 gm every eight hours. This analgesic regimen was continued for 72 hours during the post-operative period. Later, the patient received oral analgesics in the form of a tab of paracetamol 650 mg three times daily and a Tab of pregabalin 75 mg once daily at night. The patient was made to stand and walk with the help of a walker after 24 hours of surgery with active and passive physiotherapy protocol of the hospital. The patient had adequate pain relief with a maximum numerical pain score of 3 on the day of surgery and during walking on the next two days. The patient was discharged home on the fifth postoperative day.

## Discussion

Alkaptonuria is a rare autosomal recessive disorder of tyrosine metabolism. The AKU society reported a total of 1,233 patients worldwide with a total of 160 patients from India [1]. In this condition, homogentisic acid is excessively produced. Most of the homogentisic acid in circulation is excreted via the kidneys, but eventually, it starts depositing as a blackish pigment in the collagenous tissues of the body, leading to the progressive degeneration of all affected body systems. This mainly affects cartilage, heart valves, kidneys, skin, and sclera. There is no cure available for this condition. Dark-colored urine, ochronosis, and arthritis are the three characteristic features of alkaptonuria. Its systemic manifestations resemble features of osteoarthritis, ankylosing spondylitis, and collagen vascular diseases. Alkaptonuric ochronosis can result in multiple systemic complications [5]. Valvular heart disease is common in ochronosis and can be assessed using 2D echocardiography. Our patient had mild lesions of the aortic and mitral valves. Conduction abnormalities warrant the cautious use of medications acting on the cardiac conduction system. The involvement of costal cartilages may decrease the compliance of the thoracic cage, adding to the restrictive component of existing pulmonary pathology. The use of long-term non-steroidal anti-inflammatory drugs (NSAIDs) can further hamper renal function.

Painful movement of both upper limbs at the shoulder joint because of arthritis hampered intravenous access, and only a small (22G) cannula could be used. The type of body part involvement will decide the anesthesia plan in alkaptonuria patients. Lumbar spine involvement can affect the neuraxial technique. Ultrasound guidance may be used to mark the puncture site of the spinal needle [6]. However, it may not always be possible owing to a poor sonic window, as in our case. Restricted mobility of the cervical spine and suspected difficult intubation mandates the use of a video laryngoscope [7], a very handy tool. During general anesthesia, intraoperative analgesic requirements can be met with a femoral nerve block [8]. Local infiltration of an analgesic cocktail along with continuous adductor canal block [9] helps improve pain scores in the postoperative period without affecting quadriceps power. This patient had symptoms of acid peptic disease. Therefore, NSAIDs were avoided in post-operative pain management, although renal functions were normal.

## Conclusions

The anesthesia considerations in a patient with alkaptonuria can be challenging due to the involvement of all body parts essential for the safe administration of anesthesia, including venous access, the airway, and the spine. Given the rarity of this condition, discussions on anesthetic management are infrequent in academic forums, and the formulation of guidelines remains impractical. Nevertheless, the challenges encountered by an anesthesiologist can be effectively addressed through meticulous evaluation and strategic planning.
